# Expression Profiles of α1nAChR, ERK1/2, c-FOS and Matrix Metalloproteinases Among Male Smokers with Acute Coronary Syndrome

**DOI:** 10.3390/ijms27114757

**Published:** 2026-05-25

**Authors:** Nazirah Samah, Faridah Mohd Nor, Wan Mohammad Hafiz Wan Razali, Shawal Faizal Mohamad, Beh Boon Cong, Adila A. Hamid, Azizah Ugusman, Amilia Aminuddin

**Affiliations:** 1 Department of Physiology, Faculty of Medicine, Universiti Kebangsaan Malaysia, Jalan Yaacob Latif, Bandar Tun Razak, Cheras, Kuala Lumpur 56000, Malaysiadr.azizah@hctm.ukm.edu.my (A.U.); 2 Forensic Unit, Department of Pathology, Faculty of Medicine, Universiti Kebangsaan Malaysia, Jalan Yaacob Latif, Bandar Tun Razak, Cheras, Kuala Lumpur 56000, Malaysia; 3 Department of Forensic Pathology, Faculty of Medicine, Sungai Buloh Campus, Universiti Teknologi MARA, Sungai Buloh 47000, Malaysia; 4 Cardiology Unit, Hospital Canselor Tuanku Muhriz UKM, Faculty of Medicine, Universiti Kebangsaan Malaysia, Jalan Yaacob Latif, Bandar Tun Razak, Cheras, Kuala Lumpur 56000, Malaysia

**Keywords:** acute coronary syndrome, matrix metalloproteinases, atherosclerotic plaque, peripheral blood mononuclear cells

## Abstract

Acute Coronary Syndrome (ACS) is a severe manifestation of Coronary Artery Disease (CAD) caused by the rupture of unstable atherosclerotic plaques, resulting in reduced myocardial blood flow. Smoking is a major risk factor for ACS and has been associated with increased matrix metalloproteinase (MMP) activity, which contributes to the degradation of the plaque fibrous cap. However, the molecular alterations associated with smoking in ACS remain incompletely understood. This study aimed to investigate the expression of α1nAChR, ERK1/2, and c-FOS genes, together with MMP protein levels in atherosclerotic plaque tissues and peripheral blood mononuclear cells (PBMCs) of CAD patients. A total of 41 atherosclerotic plaque samples (26 smokers, 15 non-smokers) and 180 clinical subjects [*n* = 30 per group: ACS, chronic coronary syndrome (CCS), and controls; smokers and non-smokers] were included. Gene expression of ⍺1nAChR, ERK 1/2, and c-FOS was analyzed by RT-qPCR, while protein levels of MMP-2, MMP-9, and TIMP 3 were measured using ELISA. The expression of ERK 1/2 and c-FOS were significantly higher in plaque tissues of smokers compared with non-smokers (1.671- and 1.327-fold; *p* < 0.05). In PBMCs, α1nAChR expression was higher in CCS smokers (1.383-fold), while ERK 1/2 expression was higher in ACS smokers (1.355-fold). MMP-9 levels were significantly elevated in ACS and CCS compared with controls (*p* < 0.001). In conclusion, smoking CAD patients demonstrated increased expression of α1nAChR, ERK and MMP-9, indicating smoking-associated alterations in ⍺1nAChR-ERK signaling-related biomarkers in ACS.

## 1. Introduction

Cardiovascular disease (CVD) remains the leading cause of global mortality, with coronary artery disease (CAD) representing the most prevalent subtype [1,2]. Acute coronary syndrome (ACS) is the most critical clinical manifestation of CAD and is primarily triggered by rupture of unstable atherosclerotic plaques and subsequent thrombus formation [3]. Plaque instability is a consequence of chronic vascular inflammation and extracellular matrix (ECM) degradation within the fibrous cap [4,5].

Matrix metalloproteinases (MMPs) are zinc-dependent endopeptidases that regulate ECM remodeling [6]. Among them, MMP-2 and MMP-9 play central roles in plaque destabilization. MMP-2 degrades type IV collagen, laminin, and elastin, facilitating vascular remodeling and activation of other proteases [7,8]. MMP-9, primarily released by macrophages and neutrophils, degrades type I and III collagen, a key structural component of the fibrous cap, thereby weakening plaque integrity [9]. Elevated levels of MMP-2 and MMP-9 have been consistently observed in ACS [10,11]. These findings underscore the pathological importance of enhanced proteolytic activity in ACS. MMP activity is tightly regulated by tissue inhibitors of metalloproteinases (TIMP), particularly TIMP 3, which exhibits strong affinity for ECM components and broad inhibitory activity against MMPs [12]. Reduced TIMP 3 expression has been linked to increased proteolytic imbalance and higher cardiovascular risk [13,14], suggesting that disruption of the MMP–TIMP equilibrium contributes to plaque vulnerability.

Cigarette smoking is a major modifiable risk factor for ACS, conferring nearly a twofold increased risk compared to non-smokers [15]. Smoking promotes endothelial dysfunction, oxidative stress, and inflammatory activation, thereby accelerating atherosclerosis [16,17]. Importantly, smoking has been shown to enhance MMP activity and fibrous cap degradation [18]. Nicotine, the principal bioactive component of cigarette smoke, exerts its effects through nicotinic acetylcholine receptors (nAChRs), which are widely expressed in non-neuronal cells, including endothelial cells, vascular smooth muscle cells (VSMCs), and immune cells [19]. Among nAChR subtypes, the α7nAChR has been extensively studied and is known to regulate inflammatory responses and MMP expression through signaling pathways such as NF-κB and mitogen-activated protein kinase (MAPK) [20,21]. However, comparatively less attention has been given to the α1nAChR, particularly in the context of cardiovascular disease. Emerging evidence suggests that α1nAChR may also contribute to vascular remodeling, potentially through activation of the ERK 1/2 pathway, a key component of the MAPK cascade involved in regulating gene transcription related to inflammation and ECM remodeling [22,23]. Activation of ERK 1/2 can induce transcription factors such as c-FOS, which subsequently enhance MMP gene expression [21,22]. Therefore, investigating the role of α1nAChR represents a novel aspect of this study, aiming to address an important gap in understanding its contribution to atherosclerotic progression.

Despite these insights, current mechanistic evidence is largely derived from experimental models and cultured cell lines. Data from human atherosclerotic plaque tissue and peripheral blood mononuclear cells (PBMCs) remain limited. PBMCs serve as a useful surrogate for systemic inflammatory activity, and increased MMP-9 expression has been reported in PBMCs of CAD patients [24]. However, the expression profile of the α1nAChR–ERK–c-FOS signaling axis in human ACS, particularly among male smokers, has not been fully elucidated.

Therefore, this study aimed to investigate the expression profile of α1nAChR, ERK 1/2, and c-FOS, together with MMP-2 and MMP-9 protein levels in atherosclerotic plaque tissue, PBMCs, and serum among male smokers and non-smokers with ACS. We hypothesize that smoking may alter α1nAChR-ERK signaling-related biomarkers and contribute to molecular changes linked to plaque instability in male patients with ACS.

## 2. Results

### 2.1. Research 1-Atherosclerotic Plaque

#### 2.1.1. Demographic and Clinical Characteristics of Subjects

A total of 41 subjects were included in this study, comprising 26 smokers and 15 non-smokers. Overall, no significant differences were observed between smokers and non-smokers in terms of age and the clinical characteristics assessed. The mean age of smokers was 46.7 ± 1.35 years, while non-smokers had a mean age of 49.5 ± 2.62 years. The demographic and clinical characteristics of the study participants, including age, hypertension, diabetes mellitus, dyslipidemia, and family history of coronary artery disease (CAD), are presented in Table 1.

#### 2.1.2. Gene Expression Analysis

**Figure 1:**
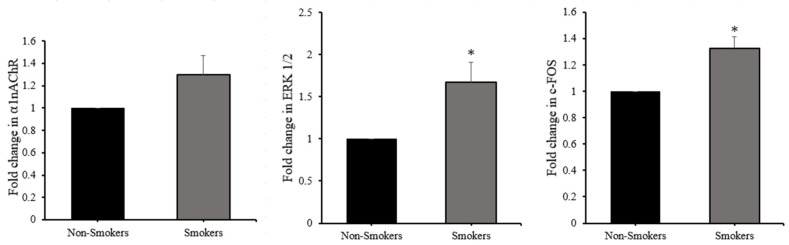
The gene expression of α1nAChR, ERK 1/2, and c-FOS in the atherosclerotic plaque of smokers and non-smokers. Values are presented as mean ± SEM. The asterisk denotes significant differences between smokers and non-smokers at * *p* < 0.05 with *n* = 26.

The gene expression of α1nAChR, ERK 1/2, and c-FOS in the atherosclerotic plaque of smokers and non-smokers is presented in Figure 1. In this study, α1nAChR gene expression showed no significant difference between smokers and non-smokers. However, both ERK 1/2 and c-FOS expression levels were significantly higher in smokers compared with non-smokers (*p* < 0.05). ERK 1/2 expression increased by 1.671-fold, while c-FOS expression increased by 1.327-fold in the smoker group.

### 2.2. Research 2-PBMCs and Serum

#### 2.2.1. Demographic and Clinical Characteristics of Patients

A total of 180 patients were successfully recruited in this study, with 30 patients in each group: ACS Smokers, ACS Non-Smokers, CCS Smokers, CCS Non-Smokers, Control Smokers, and Control Non-Smokers. Several demographic and clinical characteristics differed significantly across groups (Table 2). Age differed significantly (*p* = 0.001), with CCS non-smokers older than control non-smokers (60.87 vs. 50.77 years, *p* < 0.05). Hypertension prevalence also differed (*p* < 0.001), with CCS patients showing higher rates than controls in both smokers (20 vs. 12, *p* < 0.05) and non-smokers (27 vs. 11, *p* < 0.001). No significant differences were noted for diabetes mellitus. Dyslipidemia prevalence varied across groups (*p* = 0.002), with higher rates in CCS compared with ACS patients in both smokers (20 vs. 10, *p* < 0.05) and non-smokers (24 vs. 12, *p* < 0.05). Conversely, a family history of CAD was more common in control groups than CCS groups (Smokers: 11 vs. 3, *p* < 0.05; Non-smokers: 10 vs. 3, *p* < 0.05). Besides, Table 3 summarizes the use of cardiovascular-related medications. No significant differences were observed except for cholesterol-lowering drugs (*p* < 0.05). Among smokers, more control subjects used cholesterol-lowering medication compared with ACS patients (18 vs. 10, *p* < 0.05). Similarly, in non-smokers, control subjects used cholesterol-lowering drugs more than ACS (23 vs. 12, *p* < 0.05) and CCS (23 vs. 14, *p* < 0.05) patients. Given its role as a key cardiovascular risk factor, cholesterol-lowering medication was included as a covariate alongside age, hypertension, dyslipidemia, and family history of CAD in subsequent analyses [25,26].

#### 2.2.2. Gene Expression Analysis

**Figure 2:**
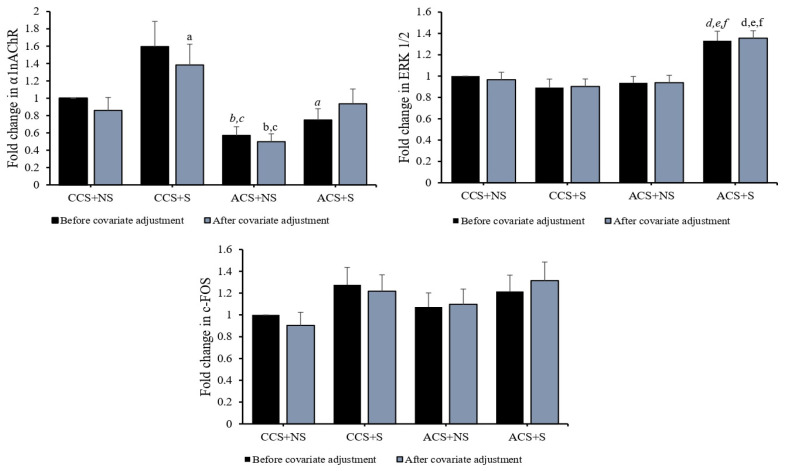
The gene expression of α1nAChR, ERK 1/2, and c-FOS in the PBMCs of ACS and CCS groups according to smoking status, before and after covariate adjustment. Values are presented as mean ± SEM with *n* = 30. *^a^ p* = 0.040 ACS + S vs. CCS + S; *^b^ p* = 0.004 ACS + NS vs. CCS + S; *^c^ p* = 0.026 ACS + NS vs. CCS + NS; ^a^ *p* = 0.047 CCS + S vs. CCS + NS; ^b^ *p* = 0.003 ACS + NS vs. CCS + S; ^c^ *p* = 0.027 ACS + NS vs. CCS + NS; *^d^ p* < 0.001 ACS + S vs. CCS + NS; *^e^ p* < 0.001 ACS + S vs. CCS + S; *^f^ p* < 0.001 ACS + S vs. ACS + NS; ^d^ *p* < 0.001 ACS + S vs. CCS + NS; ^e^ *p* < 0.001 ACS + S vs. CCS + S; ^f^ *p* < 0.001 ACS + S vs. ACS + NS.

Figure 2 shows the gene expression of α1nAChR, ERK 1/2, and c-FOS in PBMCs of ACS and CCS groups, according to smoking status, before and after covariate adjustment.

(i) *⍺1nAChR*

α1nAChR gene expression differed significantly among the study groups before covariate adjustment (*p* = 0.003) and remained significant after adjustment (*p* < 0.001), indicating that the differences were independent of potential confounders. Parameter estimate analysis after covariate adjustment showed that α1nAChR expression was significantly higher in CCS Smokers, with a 1.383-fold increase compared with CCS Non-Smokers (*p* = 0.047). In contrast, α1nAChR expression was significantly lower in ACS Non-Smokers (0.499-fold) compared with CCS Non-Smokers (*p* = 0.027).

(ii) *ERK 1/2*

ERK 1/2 gene expression differed significantly among the study groups before covariate adjustment (*p* < 0.001) and remained significant after adjustment (*p* < 0.001), indicating that the differences were independent of potential confounders. Parameter estimate analysis after covariate adjustment showed that ERK 1/2 expression was significantly higher in ACS Smokers, with a 1.355-fold increase compared with CCS Non-Smokers (*p* < 0.001). ERK 1/2 expression in ACS Smokers was also significantly higher than in ACS Non-Smokers and CCS Smokers (*p* < 0.001).

(iii) *c-FOS*

c-FOS gene expression did not differ significantly among the study groups before covariate adjustment (*p* = 0.497) and remained non-significant after adjustment (*p* = 0.187).

#### 2.2.3. Protein Levels Analysis

**Figure 3:**
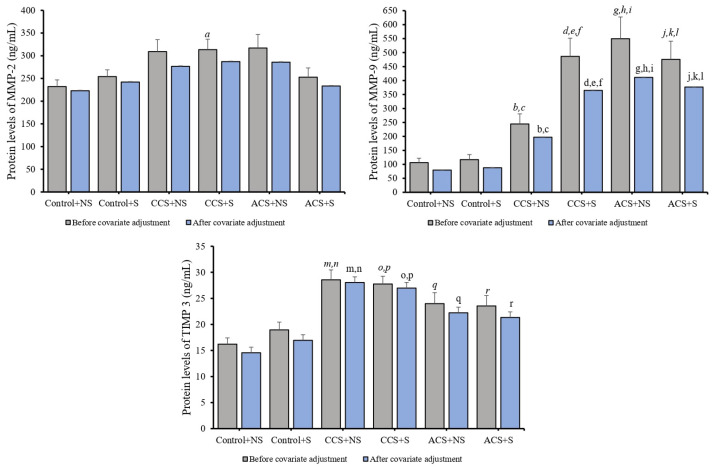
Mean serum protein levels of MMP-2, MMP-9 and TIMP 3 in the ACS, CCS, and Control groups according to smoking status, before and after covariate adjustment. Values are presented as mean ± SEM with *n* = 30. *^a^ p* = 0.036 CCS + S vs. Controls + NS; *^b^ p* < 0.001 CCS + NS vs. Controls + NS; *^c^ p* < 0.001 CCS + NS vs. Controls + S; *^d^ p* < 0.001 CCS + S vs. Controls + NS; *^e^ p* < 0.001 CCS + S vs. Controls + S; *^f^ p* = 0.022 CCS + S vs. CCS + NS; *^g^ p* < 0.001 ACS + NS vs. Controls + NS; *^h^ p* < 0.001 ACS + NS vs. Controls + S; *^i^ p* = 0.004 ACS + NS vs. CCS + NS; *^j^ p* < 0.001 ACS + S vs. Controls + NS; *^k^ p* < 0.001 ACS + S vs. Controls + S; *^l^ p* = 0.015 ACS + S vs. CCS + NS; ^b^ *p* < 0.001 CCS + NS vs. Controls + NS; ^c^ *p* = 0.004 CCS + NS vs. Controls + S; ^d^ *p* < 0.001 CCS + S vs. Controls + NS; ^e^ *p* < 0.001 CCS + S vs. Controls + S; ^f^ *p* = 0.036 CCS + S vs. CCS + NS; ^g^ *p* < 0.001 ACS + NS vs. Controls + NS; ^h^ *p* < 0.001 ACS + NS vs. Controls + S; ^i^ *p* = 0.005 ACS + NS vs. CCS + NS; ^j^ *p* < 0.001 ACS + S vs. Controls + NS; ^k^ *p* < 0.001 ACS + S vs. Controls + S; ^l^ *p* = 0.035 ACS + S vs. CCS + NS; *^m^ p* < 0.001 CCS + NS vs. Controls + NS; *^n^ p* < 0.001 CCS + NS vs. Controls + S; *^o^ p* < 0.001 CCS + S vs. Controls + NS; *^p^ p* < 0.001 CCS + S vs. Controls + S; *^q^ p* = 0.004 ACS + NS vs. Controls + NS; *^r^ p* = 0.010 ACS + S vs. Controls + NS; ^m^ *p* < 0.001 CCS + NS vs. Controls + NS; ^n^ *p* < 0.001 CCS + NS vs. Controls + S; ^o^ *p* < 0.001 CCS + S vs. Controls + NS; ^p^ *p* < 0.001 CCS + S vs. Controls + S; ^q^ *p* = 0.002 ACS + NS vs. Controls + NS; ^r^ *p* = 0.005 ACS + S vs. Controls + NS.

Figure 3 shows the mean serum protein levels of MMP-2, MMP-9 and TIMP-3 in the ACS, CCS, and Control groups according to smoking status, before and after covariate adjustment.

(i) *MMP-2*

MMP-2 protein levels differed significantly among the study groups before covariate adjustment (*p* = 0.021) but lost significance after adjustment (*p* = 0.084). However, a trend toward higher MMP-2 levels was observed in the ACS and CCS groups compared with the control group, although this increase was not statistically significant. A lower MMP-2 level was also observed in ACS Smokers.

(ii) *MMP-9*

MMP-9 protein levels differed significantly among the study groups before covariate adjustment (*p* < 0.001) and remained significant after adjustment (*p* < 0.001). After covariate adjustment, smokers within the CCS group had significantly higher MMP-9 levels than non-smokers (364.75 ± 1.2 vs. 197.24 ± 1.2 ng/mL, *p* = 0.036). In both smokers and non-smokers, MMP-9 levels were significantly higher in the ACS and CCS groups compared with controls (*p* < 0.001). Among non-smokers, MMP-9 levels were also significantly higher in ACS than in CCS (411.15 ± 1.2 vs. 197.24 ± 1.2 ng/mL, *p* = 0.005).

(iii) *TIMP-3*

TIMP-3 protein levels differed significantly among the study groups before covariate adjustment (*p* < 0.001) and remained significant after adjustment (*p* < 0.001). After covariate adjustment, CCS patients among smokers showed significantly higher TIMP 3 levels compared with controls (26.98 ± 1.1 vs. 16.94 ± 1.1 ng/mL, *p* < 0.001). Meanwhile, among non-smokers, TIMP 3 levels were significantly higher in both ACS (22.23 ± 1.1 ng/mL, *p* = 0.002) and CCS (28.05 ± 1.1 ng/mL, *p* < 0.001) compared with controls (14.55 ± 1.1 ng/mL).

## 3. Discussion

### 3.1. Research 1-Atherosclerotic Plaque

In this study, α1nAChR gene expression did not differ significantly between smokers and non-smokers in atherosclerotic plaque tissue. This suggests that smoking may not induce detectable transcriptional changes in α1nAChR at the advanced stage of disease observed in patients with ACS. At this stage, pathological processes such as inflammation, oxidative stress, endothelial dysfunction, and extracellular matrix degradation are dominant and may mask subtle changes in α1nAChR expression [27,28]. In contrast, ERK 1/2 and c-FOS expression levels were significantly higher in smokers, suggesting smoking-associated molecular alterations in ERK-cFOS signaling. Previous studies have reported that ERK 1/2 may be activated through the Ras–Raf–MEK–ERK cascade and can be influenced by nicotine and other toxic compounds present in cigarette smoke. Activated ERK 1/2 has also been associated with the regulation of transcription factors such as c-FOS [29,30,31]. c-FOS forms part of the activator protein-1 (AP-1) complex, which is involved in regulating genes associated with extracellular matrix remodeling, including MMP-2 and MMP-9 [32,33]. Alterations in this signaling pathway have been associated with vascular smooth muscle cell activity and extracellular matrix degradation, processes linked to plaque progression and instability [21,34]. Overall, although the increase in ERK 1/2 and c-FOS expression among smokers was modest, these findings may indicate smoking-associated changes in ERK-cFOS signaling-related biomarkers in coronary plaque instability.

### 3.2. Research 2-PBMCs and Serum

#### 3.2.1. Gene Expression Analysis

(i) *⍺1nAChR*

Results showed that α1nAChR expression remained statistically significant after covariate adjustment, suggesting that the observed expression differences were not substantially influenced by the included confounding variables. In detail, α1nAChR expression was significantly higher in CCS smokers compared with CCS non-smokers. This finding suggests that chronic nicotine exposure may be associated with increased α1nAChR expression in patients with CCS. Nicotine is known to interact with non-neuronal nAChRs expressed in endothelial cells, vascular smooth muscle cells (VSMC), and immune cells within the vascular wall [35]. Activation of these receptors increases intracellular Ca^2+^ influx and triggers downstream signaling pathways, including ERK signaling [36,37,38]. ERK activation promotes VSMC proliferation, migration, and MMP production, all of which contribute to atherosclerotic plaque development [18,39]. These findings highlight potential subtype-specific differences in nAChR signaling in cardiovascular disease. While the α7nAChR signaling pathway has been more extensively associated with MMP expression and inflammatory responses [20,21], the altered α1nAChR expression observed in this study suggests a potentially novel association between α1nAChR and the ERK signaling-related molecular changes in smoking-associated CAD. However, the present study did not directly assess pathway activation or downstream functional effects.

Interestingly, α1nAChR expression was significantly lower in ACS non-smokers compared with CCS non-smokers. This reduction may be attributed to the heightened inflammatory and oxidative stress environment in ACS, which can lead to receptor desensitization, internalization, and degradation, thereby altering nAChR expression and function [40,41,42,43,44]. Overall, these findings suggest that α1nAChR expression may be influenced by both smoking exposure and disease status, indicating a complex interplay between smoking, receptor regulation, and atherosclerotic progression. Although the magnitude of gene expression changes in α1nAChR was modest, these findings may reflect smoking-associated molecular alterations related to ERK signaling and plaque instability. Further mechanistic and functional studies are required to clarify the biological significance of α1nAChR-related signaling in smoking-associated plaque instability, which may subsequently provide new insights into potential targeted therapeutic interventions and biomarker development for ACS risk stratification [45].

(ii) *ERK 1/2*

Results also showed that ERK 1/2 expression remained statistically significant after covariate adjustment, suggesting that the observed expression differences were not substantially influenced by the included confounding variables. In detail, ERK 1/2 expression was significantly higher in ACS smokers compared with CCS non-smokers. ERK 1/2 expression in ACS smokers was also significantly higher than in ACS non-smokers and CCS smokers. These findings suggest smoking-associated alterations related to ERK/MAPK signaling in ACS. Previous studies have reported that nicotine exposure may influence ERK 1/2 phosphorylation in VSMC and endothelial cells, processes associated with cellular proliferation, migration, and endothelial dysfunction [37,39,46]. In addition, the inflammatory environment present in ACS may further contribute to altered ERK 1/2-related signaling. Pro-inflammatory cytokines, such as TNF-α, have been associated with increased MMP-9 expression through ERK-related signaling pathways [47]. Nicotine exposure has also been reported to influence MMP-2 and MMP-9 expression through ERK 1/2-related mechanisms, while inhibition of ERK signaling has been associated with reduced MMP production [18]. Overall, the modest elevation of ERK 1/2 gene expression observed in ACS smokers may reflect effects of smoking in molecular changes related to ERK/MAPK signaling. Targeting the ERK/MAPK signaling cascade may provide a therapeutic strategy to attenuate vascular inflammation and plaque destabilization. Pharmacological inhibition of upstream MAPK components, such as MEK1/2 inhibitors, has been shown to suppress ERK activation and reduce inflammatory and proliferative signaling in various pathological conditions [48,49].

(iii) *c-FOS*

c-FOS gene expression remained non-significant both before and after adjustment, indicating that c-FOS-related transcriptional activity may not be substantially altered in the present study population. Accordingly, no statistically significant differences in c-FOS expression were observed among the study groups. c-FOS is an immediate-early gene that is rapidly and transiently induced in response to stimuli such as oxidative stress and inflammation [50]. Its expression typically increases within minutes after stimulation, peaks within about one hour, and returns to baseline within a few hours [51,52]. Due to this short-lived expression pattern, measurement at a single time point may not capture transient changes in c-FOS levels, which could explain the absence of significant differences observed in this study.

#### 3.2.2. Protein Levels Analysis

(i) *MMP-2*

MMP-2 protein levels were statistically significant before covariate adjustment, but lost significance after adjustment. This finding suggests that the observed differences in MMP-2 levels may have been partially influenced by clinical and demographic factors included in the adjusted model. Given the established influence of these cardiovascular risk factors on inflammatory [53,54], covariate adjustment may have reduced the independent effect of smoking status on MMP-2 protein level. Consequently, no statistically significant differences in MMP-2 protein levels were observed among the study groups after adjustments. However, a trend toward higher MMP-2 levels was observed in both ACS and CCS compared with controls, although the differences were not statistically significant. Lower MMP-2 levels were also noted in ACS smokers. These findings are consistent with previous studies reporting inconsistent results regarding MMP-2 levels in CAD [55]. Some studies reported higher MMP-2 levels in patients with ACS compared with stable disease and healthy controls [56,57], whereas others found no significant differences or even lower levels following myocardial infarction [58,59]. Overall, MMP-2 levels across groups in this study may reflect the heterogeneous and context-dependent role of MMP-2 in inflammation and cardiovascular disease progression.

(ii) *MMP-9*

Results showed that MMP-9 protein levels remained statistically significant after covariate adjustment, indicating that the observed differences were not substantially influenced by the included confounding variables. Within the CCS group, smokers exhibited significantly higher MMP-9 levels compared with non-smokers, supporting a potential smoking-related upregulation of MMP-9 in patients with stable CAD. Previous studies have similarly reported elevated MMP-9 levels in smokers, with levels correlating with smoking intensity [60,61]. Nicotine can activate the MAPK/ERK signaling pathway through nicotinic acetylcholine receptors, promoting MMP-9 expression in macrophages and VSMC via AP-1 and NF-κB transcription factors [21,62,63]. In addition, MMP-9 levels were significantly higher in both ACS and CCS compared with controls among smokers and non-smokers. This indicates that MMP-9 plays a role in both stable and unstable phases of CAD. Elevated MMP-9 in CCS likely reflects ongoing inflammation and plaque development, whereas higher levels in ACS correspond to the acute inflammatory phase associated with plaque rupture (10,55,63). Furthermore, among non-smokers, MMP-9 levels were significantly higher in ACS compared with CCS, supporting its key role in plaque destabilization. MMP-9 degrades extracellular matrix components such as type IV collagen and gelatin, leading to thinning of the fibrous cap and increased susceptibility to plaque rupture [64,65]. Consistent with this mechanism, higher MMP-9 expression has been observed in macrophage-rich regions of vulnerable plaques, and patients with ruptured plaques exhibit markedly higher MMP-9 levels than those without rupture [66]. Overall, the marked elevation of MMP-9 in ACS and CCS highlights its role as a marker of inflammation and plaque instability. Higher levels in CCS smokers further suggest that nicotine exposure may enhance MMP-9 activity and contribute to atherosclerotic progression.

(iii) *TIMP-3*

Results showed that TIMP-3 protein levels remained statistically significant after covariate adjustment, indicating that the observed differences were not substantially influenced by the included confounding variables. Among smokers, CCS patients showed significantly higher TIMP-3 levels compared with controls. This increase may reflect a systemic response to enhanced ECM degradation in CAD associated with chronic nicotine exposure. TIMP 3 is a unique inhibitor of matrix metalloproteinases that is primarily bound to the ECM, enabling local regulation of proteolytic activity [67,68,69]. Nicotine-induced oxidative stress and inflammation can increase MMP activity, particularly MMP-9, accelerating ECM degradation and stimulating the release of TIMP-3 into circulation [12,69,70]. Among non-smokers, TIMP 3 levels were also significantly higher in both ACS and CCS compared with controls, indicating that inflammatory and atherosclerotic processes promote ECM degradation even in the absence of nicotine exposure. This pattern reflects an imbalance between MMP and TIMP, which may contribute to extracellular matrix breakdown, fibrous cap thinning, and plaque vulnerability [68,71]. Overall, elevated TIMP 3 levels in CCS smokers and in ACS and CCS non-smokers likely represent a systemic response to increased proteolytic activity. The imbalance between MMPs and TIMPs may contribute to persistent inflammation, extracellular matrix remodeling, and plaque instability in CAD.

## 4. Materials and Methods

### 4.1. Patient Recruitment and Sample Collection

The study was conducted in accordance with the Declaration of Helsinki and received approval from the Centre for Research and Instrumentation Management (CRIM) of Universiti Kebangsaan Malaysia (UKM PPI/111/8/JEP-2022-112, 29 March 2022). Patient recruitment and sample collection were conducted at Hospital Canselor Tuanku Muhriz (HCTM), Universiti Kebangsaan Malaysia. Atherosclerotic plaque tissues were obtained from male cadavers aged ≥18 years during autopsy within 24 h after death. The subjects had died suddenly due to myocardial infarction, obstructed coronary arteries, coronary atherosclerosis, or coronary atheroma. Smoking status and medical history were obtained from family members, close acquaintances, or hospital records. Cigarette exposure was calculated in pack-years, where one pack-year equals smoking 20 cigarettes per day for one year [72]. The collected plaque tissue samples were categorized into two groups: Group 1: Smokers and Group 2: Non-smokers. Appendix A showed the detailed inclusion criteria for the subjects. From each subject, three plaque tissue samples were collected, preserved in RNAlater, and stored at −80 °C until analysis.

For blood samples, venous blood was collected from male patients aged ≥ 18 years undergoing computed tomography (CT) or coronary angiography for suspected or diagnosed coronary artery disease (CAD). Clinical information, smoking status, and medication history were obtained from patients or hospital records, and smoking exposure was calculated in pack-years. Patients were categorized into six groups: Group 1: ACS Smokers, Group 2: ACS Non-Smokers, Group 3: CCS Smokers, Group 4: CCS Non-Smokers, Group 5: Control Smokers, and Group 6: Control Non-Smokers. Appendix B showed the detailed inclusion criteria for the patients. From each patient, a total of 20 mL of venous blood was collected into EDTA and serum tubes, stored at 4–8 °C, and processed within 24 h for peripheral blood mononuclear cell (PBMC) and serum isolation. PBMCs were preserved in RNAlater, and both PBMC and serum samples were stored at −80 °C until further analysis.

### 4.2. PBMC Isolation and Serum Collection

PBMCs were isolated from whole blood using Histopaque-1077 (Sigma-Aldrich, St. Louis, MO, USA) by density gradient centrifugation according to the manufacturer’s protocol [73]. Briefly, 4 mL of whole blood was layered onto 4 mL Histopaque-1077 in a 15 mL centrifuge tube and centrifuged at 400× *g* for 30 min at room temperature with low acceleration and brake. The PBMC layer at the plasma–Histopaque interface was carefully collected and transferred to a new tube. Cells were washed with phosphate-buffered saline (PBS) and centrifuged at 250× *g* for 10 min, followed by a second wash under the same conditions. The final cell pellet was resuspended in 1 mL of RNAlater (Thermo Fisher Scientific, Waltham, MA, USA) and stored at −80 °C until RNA extraction. For serum collection, whole blood was centrifuged at 2000× *g* for 15 min at 4 °C. The serum was collected, aliquoted into microcentrifuge tubes, and stored at −80 °C for subsequent protein analysis.

### 4.3. Gene Expression of α1nAChR, ERK 1/2, and c-FOS in Atherosclerotic Plaque Tissue and PBMC Using Quantitative Real-Time Polymerase Chain Reaction (RT-qPCR)

#### 4.3.1. RNA Extraction

Total RNA from atherosclerotic plaque tissue was extracted using the RNeasy Lipid Tissue Mini Kit (QIAGEN, Germantown, MD, USA) according to the manufacturer’s instructions [74]. Briefly, plaque tissues stored at −80 °C (40–80 mg) were homogenized in QIAzol lysis reagent using an Omni Bead Ruptor (20 Hz, 14 min). After incubation at room temperature for 5 min, chloroform was added for phase separation and centrifuged at 12,000× *g* for 15 min at 4 °C. The aqueous phase was mixed with 70% ethanol and transferred to an RNeasy spin column. The column was washed with RW1 and RPE buffers, and RNA was eluted with 30 µL nuclease-free water and stored at −80 °C until cDNA synthesis. Meanwhile, total RNA from peripheral blood mononuclear cells (PBMCs) was extracted using the Quick-RNA Mini Kit (Zymo Research, Irvine, CA USA) following the manufacturer’s protocol [75]. PBMC samples preserved in RNAlater were thawed and centrifuged to remove the storage solution. The cell pellet was lysed and homogenized using an Omni Bead Ruptor (20 Hz, 14 min). The lysate was filtered, mixed with ethanol, and loaded onto a Zymo-Spin column. After on-column DNase digestion and washing steps, RNA was eluted with 30 µL nuclease-free water and stored at −80 °C until cDNA synthesis.

#### 4.3.2. cDNA Synthesis and RT-qPCR

The purity and concentration of extracted RNA from plaque and PBMC samples were measured using a spectrophotometer. Complementary DNA (cDNA) was synthesized from total RNA using the Viva cDNA Synthesis Kit (Vivantis Technologies Sdn Bhd, Subang Jaya, Selangor, Malaysia) according to the manufacturer’s instructions [76]. Briefly, the RNA–primer mixture was prepared (Table 4) and incubated at 65 °C for 5 min using a Select Cycler II Thermal Cycler, followed by cooling on ice for 2 min. The cDNA synthesis mixture (Table 5) was then added to the RNA–primer mixture and briefly centrifuged. Reverse transcription was performed at 42 °C for 60 min, followed by enzyme inactivation at 85 °C for 5 min. The synthesized cDNA was cooled on ice and stored at −20 °C until qPCR analysis.

The qPCR was then performed using the qPCRBIO SyGreen Mix Separate-ROX kit (PCR Biosystems, London, UK) according to the manufacturer’s protocol [77]. ROX dye was first added to the 2× qPCRBIO SyGreen Mix to prepare the working mix. A master mix containing the SYBR mix and gene-specific primers was prepared for each target gene (Table 6), and 9 µL was dispensed into each well of a 96-well PCR plate. The qPCR primers were pre-validated by GeneCopoeia (GeneCopoeia, Rockville, MD, USA) with the respective accession numbers listed in Table 7 Next, 1 µL of cDNA template was added to each reaction, while nuclease-free water was used for the no-template control (NTC), giving a final reaction volume of 10 µL per well. Amplification was performed using the Bio-Rad CFX96 Real-Time System (Bio-Rad Laboratories, Hercules, CA, USA) following the cycling conditions provided in Table 8 The cycle threshold (Ct) values obtained were used to calculate relative gene expression using the 2^−ΔΔCt^ method. The non-smokers group in Research 1 (Atherosclerotic plaque) and the CCS non-smokers group in Research 2 (PBMC) were used as the calibrator groups, with their expression levels normalized to a baseline value of 1. GAPDH was used as the internal housekeeping gene.

### 4.4. Protein Quantification by ELISA

Serum levels of MMP-2, MMP-9, and TIMP-3 were quantified using human enzyme-linked immunosorbent assay kits according to the manufacturer’s instructions [78]. Serum samples were centrifuged at 1000× *g* for 20 min at 2–8 °C, and the supernatant was diluted 100-fold prior to analysis. Serial dilutions of reference standards were prepared to generate standard curves for each protein. Diluted samples and standards (100 µL) were added in duplicate to the microplate wells and incubated at 37 °C for 90 min. After incubation, biotin-labeled detection antibody was added and incubated for 60 min at 37 °C, followed by washing steps. HRP-conjugate working solution was then added and incubated for 30 min at 37 °C. After additional washes, substrate solution was added and incubated for 15 min at 37 °C, and the reaction was stopped with the stop solution. The absorbance was measured at 450 nm using a microplate reader. Protein concentrations of MMP-2, MMP-9, and TIMP 3 were calculated from the standard curves.

### 4.5. Statistical Analysis

#### 4.5.1. Gene Expression

Statistical analysis was performed using IBM SPSS Statistics version 30. Data normality was assessed using the Shapiro–Wilk test. For both tissue atherosclerotic and PBMC analyses, normally distributed data were analyzed using the Generalized Linear Model (GLM) with a normal distribution and identity link function, whereas non-normally distributed data were analyzed using a GLM with Gamma distribution and log link function. Model assumptions were checked before analysis. Statistical significance was set at *p* < 0.05, and results were expressed as mean ± standard error of the mean (SEM).

For PBMC analyses, additional covariate adjustment was performed by including age, hypertension, dyslipidemia, family history of CAD, and use of cholesterol-lowering medication in the model. These covariates were selected based on their established relevance to cardiovascular risk and potential influence on gene expression profiles [79,80] as well as the presence of statistically significant differences between study groups. All covariates were entered simultaneously into the model as fixed-effect predictors. Hypertension, dyslipidemia, family history of CAD, and medication use were entered as categorical variables, while age was included as a continuous covariate.

#### 4.5.2. Protein Quantification

Statistical analysis was performed using IBM SPSS Statistics version 30. Data normality was assessed using the Shapiro–Wilk test. Non-normally distributed data were log_10_-transformed prior to analysis. Differences in protein levels between groups were initially analyzed using one-way analysis of variance (ANOVA) without covariate adjustment. To account for potential confounding factors, analysis of covariance (ANCOVA) was subsequently performed by including age, hypertension, dyslipidemia, family history of CAD, and use of cholesterol-lowering medication as covariates. Covariates were entered into the model in the same manner as described in Section 4.5.1. Statistical significance was set at *p* < 0.05, and results are presented as mean ± standard error of the mean (SEM) on the original scale.

## 5. Conclusions

In conclusion, this study demonstrated increased expression of α1nAChR–ERK1/2 signaling-related biomarkers in atherosclerotic plaque tissue and PBMCs of smoking CAD patients. Elevated MMP-9 protein levels also suggest the possible molecular alterations associated with smoking in ACS. These findings provide preliminary evidence that α1nAChR-ERK signaling may be associated with molecular processes linked to plaque instability and ACS progression. Overall, this study contributes to a better understanding of smoking-associated molecular changes in CAD and may serve as a basis for future functional and mechanistic studies, as well as the exploration of potential targeted therapeutic strategies.

## 6. Limitations and Suggestions

This study has several limitations that should be acknowledged. First, it primarily relied on gene expression profiling, which does not directly reflect the mechanistic causality for the α1nAChR–ERK signaling pathway in CAD or ACS pathogenesis. Although differential expression of key genes and proteins was observed, the study design does not allow confirmation of pathway activation or direct downstream functional effects. Second, the relatively limited sample size, particularly in the atherosclerotic plaque study, may influence the robustness and generalizability of the findings and should be considered when interpreting the results.

To address these limitations, future studies should incorporate protein-level validation (western blotting), receptor functional assays, and pathway inhibition or stimulation experiments to confirm the biological role of the α1nAChR–ERK signaling axis. In addition, larger and well-characterized patient cohorts are warranted to validate the present findings and to strengthen statistical power. Longitudinal study designs may also help to better elucidate the temporal relationship between smoking exposure, nAChR signaling, and CAD progression. Overall, while the present study provides preliminary evidence supporting the potential involvement of α1nAChR-ERK signaling in CAD, further mechanistic investigations are required to confirm its functional role in disease development.

## Figures and Tables

**Table 1:** Demographic and clinical characteristics of Subjects.

Characteristics		Smokers N = 26	Non-Smokers N = 15	*p*-Value
Age (year)		46.7 ± 1.35	49.5 ± 2.62	0.351
Hypertension	Yes, *n* (%) No, *n* (%) Unknown, *n* (%)	6 (23.1%) 15 (57.7%) 5 (19.2%)	1 (6.7%) 11 (73.3%) 3 (20%)	0.393
Diabetes Mellitus	Yes, *n* (%) No, *n* (%) Unknown, *n* (%)	1 (3.8%) 20 (76.9%) 5 (19.2%)	2 (13.3%) 10 (66.7%) 3 (20%)	0.520
Dyslipidemia	Yes, *n* (%) No, *n* (%) Unknown, *n* (%)	0 (0%) 21 (80.8%) 5 (19.2%)	1 (6.7%) 11 (73.3%) 3 (20%)	0.406
Family history of CAD	Yes, *n* (%) No, *n* (%) Unknown, *n* (%)	0 (0%) 21 (80.8%) 5 (19.2%)	1 (6.7%) 11 (73.3%) 3 (20%)	0.183

**Table 2:** Demographic and clinical characteristics of patients.

Characteristics		ACS		CCS		Control		*p*-Value
		Smokers (*n* = 30)	Non-Smokers (*n* = 30)	Smokers *(n* = 30)	Non-Smokers (*n* = 30)	Smokers (*n* = 30)	Non-Smokers (*n* = 30)	
Age (year)		51.83 ± 1.98	56.17 ± 2.47	53.50 ± 1.98	60.87 ± 1.72 ^a^	49.03 ± 2.07	50.77 ± 2.12	0.001 *
Weight (kg)		75.23 ± 1.96	74.77 ± 2.14	74.07 ± 1.55	74.23 ± 1.87	75.90 ± 1.88	78.03 ± 2.53	0.752
BMI (kg/m^2^)		25.64 ± 0.67	26.76 ± 0.82	25.20 ± 0.54	26.41 ± 0.70	26.09 ± 0.63	26.54 ± 0.85	0.703
Heart rate (bpm)		65.77 ± 1.57	78.07 ± 2.82 ^d^	75.0 ± 2.71	78.97 ± 2.03 ^e^	70.20 ± 2.05	64.5 ± 1.70	<0.001 *
Hypertension	Yes, *n* (%)	16 (53.3%)	23 (76.7%)	20 ^f^ (66.7%)	27 ^g^ (90%)	12 (40%)	11 (36.7%)	<0.001 *
Diabetes Mellitus	Yes, *n* (%)	8 (26.7%)	14 (46.7%)	13 (43.3%)	16 (53.3%)	9 (30%)	9 (30%)	0.183
Dyslipidemia	Yes, *n* (%)	10 (33.3%)	12 (40%)	20 ^h^ (66.7%)	24 ^i^ (80%)	15 (50%)	19 (63.3%)	0.002 *
Family history of CAD	Yes, *n* (%)	5 (16.7%)	5 (16.7%)	3 (10%)	3 (10%)	11 ^h^ (36.7%)	10 ^i^ (33.3%)	0.029 *

* *p* < 0.05 significant. Data for age, weight, BMI and heart rate are presented as mean ± SEM. Data for hypertension, diabetes mellitus, dyslipidemia, and family history of CAD are presented as *n*, the number of patients. Post-hos analysis: ^a^ *p* < 0.05, CCS non-smokers vs. control non-smokers; ^d^ *p* < 0.05, CCS smokers vs. control smokers; ^e^ *p* < 0.001, CCS non-smokers vs. control non-smokers; ^f^ *p* < 0.05, CCS smokers vs. ACS smokers; ^g^ *p* < 0.05, CCS non-smokers vs. ACS non-smokers; ^h^ *p* < 0.05, control smokers vs. CCS smokers; ^i^ *p* < 0.05, control non-smokers vs. CCS non-smokers.

**Table 3:** Use of cardiovascular-related medicine.

Medication Groups		ACS		CCS		Controls		*p*-Value
		Smokers (*n* = 30)	Non-Smokers (*n* = 30)	Smokers (*n* = 30)	Non-Smokers (*n* = 30)	Smokers (*n* = 30)	Non-Smokers (*n* = 30)	
ACE inhibitors	Yes, *n* (%)	9 (30.0%)	3 (10.0%)	8 (26.7%)	13 (43.3%)	6 (20.0%)	7 (23.3%)	0.084
Angiotensin II inhibitors	Yes, *n* (%)	3 (10.0%)	3 (10.0%)	2 (6.7%)	6 (20.0%)	2 (6.7%)	5 (16.7%)	0.498
Anticoagulant agent	Yes, *n* (%)	1 (3.3%)	0 (0%)	1 (3.3%)	0 (0%)	2 (6.7%)	1 (3.3%)	0.624
Antiplatelet agent	Yes, *n* (%)	11 (36.7%)	10 (33.3%)	11 (36.7%)	6 (20.0%)	6 (20.0%)	15 (50.0%)	0.113
Beta inhibitors	Yes, *n* (%)	9 (30.0%)	8 (26.7%)	10 (33.3%)	7 (23.3%)	8 (26.7%)	11 (36.7%)	0.884
Calcium-channel inhibitors	Yes, *n* (%)	2 (6.7%)	2 (6.7%)	6 (20.0%)	5 (16.7%)	3 (10.0%)	6 (20.0%)	0.393
Cholesterol -lowering drugs	Yes, *n* (%)	10 ^α,β^ (33.3%)	12 ^δ^ (40.0%)	13 ^θ^ (43.3%)	14 ^μ^ (46.7%)	18 (60.0%)	23 (76.7%)	0.011 *

* *p* < 0.05 significant. Data are presented in *n*, the number of patients; ACE: angiotensin-converting enzyme. Post-hoc analysis: ^α^ *p* < 0.05, ACS smokers vs. control smokers; ^β^ *p* < 0.001, ACS smokers vs. control non-smokers; ^δ^ *p* < 0.05, ACS non-smokers vs. control non-smokers; ^θ^ *p* < 0.05, CCS smokers vs. control non-smokers; ^μ^ *p* < 0.05, CCS non-smokers vs. control non-smokers.

**Table 4:** RNA-primer mixture.

Component	Volume
Total RNA	1 μg
Oligo d(T)	1 μL
10 mM dNTPs	1 μL
Nuclease-free water	Varies
Total final volume	10 μL

**Table 5:** cDNA mixture.

Component	Volume
10 X M-MuLV Buffer	2.0 μL
M-MuLV Reverse Transcriptase	0.5 μL
Nuclease-free water	7.5 μL
Total final volume	10 μL

**Table 6:** qPCR master mix.

Component	Volume for One Reaction	Volume for One Target Gene per Plate
2x qPCRBIO Sygreen Mix	5.0 μL	120 μL
Targeted gen primers (2 μM)	2.0 μL	48 μL
Nuclease-free water	2.0 μL	48 μL
Total final volume	9 μL	216 μL

**Table 7:** Primers for qPCR.

Gene	Primer ID	GenBank Accession No.	PCR Size
α1nAChR	Hs-QRP-40509	NM_001039523	143
ERK 1/2	Hs-QRP-41066	NM_002745	147
c-FOS	Hs-QRP-21506	NM_005252	95
GAPDH	Hs-QRP-20169	NM_002046	152

**Table 8:** qPCR reaction protocol.

Process	Cycle	Temperature °C	Time
Polymerase enzyme activation	1	95	2 min
Denaturation	40	95	5 s
Annealing	40	60	20 s

## Data Availability

The study’s original contributions are documented within the article. For further inquiries, please contact the corresponding author directly.

## Appendix A

**Table A1:** Inclusion and Exclusion Criteria for Atherosclerotic Plaque Tissue Study.

Group	Inclusion Criteria	Exclusion Criteria
1 (Smokers)	Male (≥18 years) who died suddenly (heart attack, obstructed coronary arteries/coronary atherosclerosis/coronary atheroma) Smokers (more than 10 pack-years)	Subjects with a history of chronic diseases (lung disease/kidney disease/cancer/chronic inflammatory diseases and others)
2 (Non-Smokers)	Male (≥18 years) who died suddenly (heart attack, obstructed coronary arteries/coronary atherosclerosis/coronary atheroma) Non-smokers (less than 10 pack-years/never smoked)	Smokers (more than 10 pack-years) Subjects with a history of chronic diseases (lung disease/kidney disease/cancer/chronic inflammatory diseases and others)

## Appendix B

**Table A2:** Inclusion and Exclusion Criteria for PBMC and Serum Study.

Group	Inclusion Criteria	Exclusion Criteria
1.(ACS Smokers)	Male (≥18 years) who were diagnosed with ACS (characterized by ECG changes and elevated troponin I) Smokers (more than 10 pack-years)	Patients with a history of chronic diseases (lung disease/kidney disease/cancer/chronic inflammatory diseases and others) Patients with previous history of ACS/had previously undergone an angiogram
2 (ACS Non-Smokers)	Male (≥18 years) who were diagnosed with ACS (characterized by ECG changes and elevated troponin I) Non-smokers (less than 10 pack-years/never smoked)	Smokers (more than 10 pack-years) Patients with a history of chronic diseases (lung disease/kidney disease/cancer/chronic inflammatory diseases and others) Patients with previous history of ACS/had previously undergone an angiogram
3 (CCS Smokers)	Male (≥18 years) who were diagnosed with CCS (characterized by not ACS and >50% stenosis seen in angiogram/CT) Smokers (more than 10 pack-years)	Patients with a history of chronic diseases (lung disease/kidney disease/cancer/chronic inflammatory diseases and others) Patients with previous history of ACS/had previously undergone an angiogram
4 (CCS Non-Smokers)	Male (≥18 years) who were diagnosed with CCS (characterized by not ACS and >50% stenosis seen in angiogram/CT) Non-smokers (less than 10 pack-years/never smoked)	Smokers (more than 10 pack-years) Patients with a history of chronic diseases (lung disease/kidney disease/cancer/chronic inflammatory diseases and others) Patients with previous history of ACS/had previously undergone an angiogram
5 (Control Smokers)	Male (≥18 years) with no ACS and <50% stenosis seen in angiogram/CT Smokers (more than 10 pack-years)	Patients with a history of chronic diseases (lung disease/kidney disease/cancer/chronic inflammatory diseases and others) Patients with previous history of ACS/had previously undergone an angiogram
6 (Control Non-smokers)	Male (≥18 years) with no ACS and <50% stenosis seen in angiogram/CT Non-smokers (less than 10 pack-years/never smoked)	Smokers (more than 10 pack-years) Patients with a history of chronic diseases (lung disease/kidney disease/cancer/chronic inflammatory diseases and others) Patients with previous history of ACS/had previously undergone an angiogram
